# Near infrared spectroscopy monitoring during carotid endarterectomy in elderly patients

**DOI:** 10.1186/1471-2318-11-S1-A57

**Published:** 2011-08-24

**Authors:** A Scolaro, F Di Stefano, C Ramondetta, A Lomeo

**Affiliations:** 1 AO per l’emergenza Cannizzaro-Catania, Italy

## Background

Various modalities are used for cerebral monitoring during carotid endarterectomy (CEA). The aim of this study was to evaluate the accuracy of transcranial cerebral oximetry (TCO ) for monitoring cerebral ischemia during carotid cross-clamping.

## Materials and methods

193, consecutive patients, 101 males, mean age 72 years, underwent CEA in our Institution from September 2007 to December 2009. All operations were only performed by two surgeons and all patients were monitored with TCO during general anesthesia. TCO parameters were always registered in: base-line, anesthesia induction, carotid clamping and declamping, wake up and estubation. The relationship with TCO was described in terms of the decrease in percentage oxygenation. Standard endarterectomy was performed through a longitudinal arteriotomy. The majority of arteriotomies were sutured with a dacron patch. Primary suturing was done only when it was possible to use the eversion technique.

## Results

CEA was completed successfully in all patients. The mean duration of cross-clamping of the carotid artery was 18 minutes. No major or minor strokes occurred and a shunt was never placed. Minor complications were 7 neck hematomas, 3 patients with hoarseness that cleared up and 1 patient with neuralgia over the operative scar that subsequently cleared up.

## Conclusions

The principal goal of monitoring during CEA is to identify possible causes of neurological impairment sufficiently early to allow prompt correction of the cause. Mental status evaluation during carotid cross-clamping while the patient is awake remains the gold standard with which other methods of monitoring should be compared. Under general anesthesia, monitoring with EEG remains the gold standard. In our study of 193 patients TCO was evaluated under general anesthesia, for detecting cerebral ischemia during cross-clamping of the carotid artery for CEA. TCO showed a decrease greater than 20% in 24 patients. In all these cases some maneuvers by anesthesiologists were made as hypertension and hypercapnia (40-45 mmHg) induction to restore the TCO normal range . In this case a shunt was never placed and no neurological problems occurred. We believe that under general anesthesia, TCO is a practical and non-invasive monitoring system with high sensitivity.

